# Determining the Instructional Effectiveness of Online Vaccine Education Modules: A Focus-Group Analysis

**DOI:** 10.15694/mep.2019.000215.1

**Published:** 2019-11-26

**Authors:** Sarah Williams, Shannon Clark, Sharon Humiston, Barbara Pahud, Donald Middleton, Kadriye O. Lewis

**Affiliations:** 1 Vanderbilt University Medical Center; 2 Children's Mercy Hospital; 3 University of Pittsburgh Medical Center

**Keywords:** Resident education, vaccine education, focus group, Cognitive Load Theory, CLT, Attention, Relevance, Confidence, Satisfaction Model, ARCS, Internet Based Learning, IBL

## Abstract

This article was migrated. The article was marked as recommended.

Vaccine education for pediatric and family medicine residents is inadequate. Implementation of evidence-based instructional design methods for medical education is understudied. We conducted four focus groups with residents who had completed a novel immunization curriculum to explore their satisfaction with design, content and impact on confidence. Data were analyzed using thematic content analysis. Overall satisfaction with the curriculum was high. Residents valued the interactive design and content, reported improvement in confidence in discussing vaccines with parents, and shared recommendations for future iterations of the modules. Technical challenges were reported with the learning management system. Medical education modules developed using best practices in instructional design were well-liked by trainees and future modules should be developed using these principles.

## Introduction

Childhood vaccination is an effective method to reduce morbidity and mortality. However, vaccination rates in the U.S remain below goals. ( Office of Disease Prevention and Health Promotion, 2018) Strong recommendations from healthcare providers have been shown to positively impact vaccination decisions ( Opel *et al.*, 2013), yet, immunization and communication education during pediatric residency training is inadequate.( Williams and Swan, 2014) To address this deficiency, we created the Collaboration for Vaccine Education and Research (CoVER) to augment residents’ immunization knowledge and vaccine communication competency. A 4-module curriculum was developed using empirically supported instructional design principles based on Cognitive Load Theory (CLT) ( Sweller, 2010), Multimedia Learning (MML) ( Mayer, 2009), and the Attention, Relevance, Confidence, Satisfaction Model (ARCS) ( Keller, 2010). We delivered the CoVER curriculum (“CoVER”) using a flipped-classroom approach and Internet Based Learning (IBL), which has been associated with greater positive effects in learning compared to traditional or non-IBL educational techniques. ( Wittich *et al.*, 2017)

As there is little data evaluating the instructional effectiveness of flipped-classroom technology in training programs ( Cook *et al.*, 2010), this study seeks to address this deficit through evaluation of the instructional effectiveness of the CoVER curriculum.

## Methods

This qualitative study used focus group interviews to obtain pediatric and family medicine residents’ (“residents”) educational experience with the four CoVER modules. The study was exempt by the Institutional Review Board at Children’s Mercy, Kansas City. Implied consent was obtained through voluntary focus group participation.

### Study Setting and Participants

In Fall 2018 we recruited residents for focus groups from a convenience sample of four residency programs that received CoVER during a 1-year pilot program. Participants included only those who had completed >1-of-4 CoVER modules. Recruitment was conducted via e-mail, through residency program announcements, or during resident conferences. No monetary incentives were provided.

### CoVER Curriculum

Five experts in pediatric infectious disease, immunization, immunization education, residency education, and adult learning developed four 1-hour modules incorporating best practices in instruction design principles. We applied the ARCS Model to promote learning and sustain motivation in the learning process, and incorporated principles of MML and CLT to manage intrinsic load (working memory load) and maximize storage of new knowledge in long-term memory.

CoVER focused on key aspects of vaccines and vaccination (vaccine fundamentals, vaccine preventable diseases, vaccine safety, communication) and was piloted July 2017 through June 2018.

### Instrument

The focus group moderator’s guide included thirteen open and closed format questions focusing on instructional content and design, the learning platform’s usability, and suggestions for improvement. Clarifying questions were utilized when needed. Because our primary goal was to evaluate instructional effectiveness, questions were grounded in these concepts.

### Data Collection and Analysis

Group interviews were conducted at each institution and facilitated by a moderator. Focus groups were audio recorded and transcribed for review and analysis. Two coders read the transcripts multiple times to gain a sense of the entire context of material and to discover quotes and examples to illuminate key concepts and themes. A list of codes was developed prior to review using the focus group guide and then applied to quotes within transcripts. Coded responses were grouped into thematic domains. Additional codes were developed during review to capture additional domains as needed. Data were analyzed using thematic content analysis and open, axial, and selective coding procedures from grounded theory principles. Differences in coder interpretations were resolved by discussions leading to consensus, or the inclusion of a third coder. Charts and tabulations were used to reduce researcher bias.

## Results/Analysis

The study cohort comprised 28 residents representing all years of training. One focus group was conducted at each of four academic institutions: Vanderbilt University Medical Center, Children’s Mercy, Kansas City, University of Kansas Medical Center, and Truman Medical Center. Identified reflections were clustered into **six main themes.**

**1. *Content Design***

Respondents reported that the material was well planned and clear, sharing that CoVER content “wasn’t the same thing” as every other required training. Residents valued the broad range of content with many specifying the importance of learning about public and/or patient vaccine misinformation, parental concerns, “odd ball” or out-of-the-ordinary vaccine questions, negative media stories, delayed vaccination schedules, and vaccine components. Additionally, residents responded positively to learning about vaccine “basics” and resources for additional information. One resident shared:

“What we would want in a vaccine module is pretty much what you guys gave us.”

2. ***Module Structure/Learning Engagement***

Residents liked the content’s logical sequencing with relevant “small chunks” of information and the variety of interactive activities such as flashcards, simple games, knowledge checks, and multimedia integration of content (videos and links). Respondents believed that the content was “very visually appealing”, “fairly simple”, and presented in a “user-friendly” manner.

3. ***Perceived Learning and Confidence***

Most participants reported the modules greatly improved their own confidence in their vaccine knowledge and comfort in discussing and recommending vaccines. One resident recalled utilizing new knowledge to convince a previously vaccine hesitant parent to vaccinate her child. Several residents shared that modules were helpful to “prepare for concerns from parents.” One resident described the impact on his confidence as now:

“Being able to ask them why they’re hesitant and be more confident that I’ll have an answer to whatever reason.”

4. ***Challenges***

Several participants reported technology challenges with the Learning Management System (LMS) that housed CoVER, such as difficulty with logging out, losing prior work, or being uncertainty if they had completed a module:

“That was the only thing I didn’t like about it. I didn’t know if I was done.”

5. ***Recommendations for the Future***

Several ideas were presented for future iterations of CoVER (Table1). Suggestions included broadening content, changing the LMS, and adding new access options.

**Table 1.** Residents’ Recommendations for Future Iterations of CoVER

**Content** • More direct examples of common myths or concerns • More on how to respond to parents who have concerns • More on vaccine side effects and counselling families following a reaction • More on reporting vaccine reactions **LMS** • More frequent reminders to complete modules • Quick links for accessing modules • Clear visual prompts to verify completed modules **Supplementary Resources** • CoVER website for parents • Downloadable summaries of material • More frequent vaccine instruction (e.g., module of the month)

6. ***Overall Satisfaction***

Residents had high overall satisfaction with CoVER. Several participants reported the modules were the best they had completed in training thus far. Almost all participants agreed the modules incorporated a clear, clean layout and visual design. They found the modules user-friendly and valued the interactive nature and embedded resources.

## Discussion

This qualitative study provides insights into the effectiveness of the methodologies and theory-driven instructional design principles utilized to create CoVER. Results support that well-designed modules using best practices in instructional design (CLT, MML, ARCS) produce higher learning satisfaction and positive impact on learners. Residents verbalized benefits from 1) not being exposed to extraneous material (CLT, MML), 2) visual signals for essential material (MML, ARCS), 3) interesting and appropriate graphics to limit redundancy and text (MML, CLT, ARCS), and 4) appropriate spacing of new information (CLT, MML). Further, the content presented using these strategies positively impacted the perceived confidence in discussing vaccines with parents or patients with concerns (ARCS), a great challenge for healthcare providers currently.

Despite overall satisfaction with the modules, technical challenges were identified by several participants with the LMS. Participants were at times frustrated with logging in/out of the LMS and confirming module completion, which may have impacted completion rates of modules. Participants also reported several suggestions to improve or expand future CoVER training. These concerns and suggestions will be addressed in future iterations of CoVER.

Limitations include the limited sample size, although sufficient for our purposes, and not all participants completed all 4 modules. The study could also be affected by participation and/or social bias. However, we achieved thematic saturation early during data analysis and, thus believe that the themes reported herein are likely representative of majority of participants’ views.

## Conclusion

A vaccine education curriculum for pediatric and family medicine residents developed using evidence-based instructional design was well received. Future modules for medical residents should be developed using these principles.

## Take Home Messages

• The CoVER curriculum, developed using best practices in instructional design, was well received by pediatric and family medicine residents.
• Technical challenges with the learning management system represent opportunities for curriculum improvement.

## Notes On Contributors

Sarah Williams MD, MPH: Assistant Professor of Pediatrics in the Department of Pediatrics at Vanderbilt University Medical Center in Nashville, Tennesse. Her research focuses on pediatric immunization and medical education across the spectrum of lifelong learners.

Shannon Clark MPH: Program Director for CoVER in the Department of Pediatrics at Children’s Mercy Hospital in Kansas City, Missouri.

Barbara Pahud MD, MPH: Associate Professor of Pediatric Infectious Disease in Department of Pediatrics at Children’s Mercy Hospital in Kansas City, Missouri. She is a national leader in vaccine-related research.

Sharon Humiston MD, MPH: Professor of Pediatrics at Children’s Mercy Hospital in Kansas City with extensive expertise in immunization-related research and provider education.

Donald Middleton MD: Professor of Family Medicine at the University of Pittsburgh Medical Center. He has dedicated his career to advocating and teaching prevention medicine.

Kadriye O. Lewis Ed.D: Professor and Director of Evaluation and Program Development in the Department of Pediatrics at Children’s Mercy Hospital in Kansas City, Missouri with extensive experience in developing, implementing, and evaluating education techniques and curricula for medical providers.

## Declarations

All authors received funding from the Pfizer Foundation through an Independent Grant for Learning & Change. KOL - No additional conflicts BAP - Clinical investigator on trials funded by GlaxoSmithKline, Pfizer, and Alios Biopharma/Janssen and has received honoraria from Pfizer, Sequirus, and Sanofi Pasteur for service on advisory boards and nonbranded presentations. DM - Advisory boards for Pfizer, GlaxoSmithKline, and Sanofi Pasteur, lectures for Pfizer and Seqirus, co-principal investigator for a CDC grant. SEW - Additional grant funding from the American Academy of Pediatrics and Pfizer for an Independent Grant for Learning & Change SGH - Consultant to Immunization Action Coalition (nonprofit organization) and Sanofi Pasteur.
