# Supplementary material for: Determining the Instructional Effectiveness of Online Vaccine Education Modules: A Focus-Group Analysis
Source: MedEdPublish (2016). 2019 Nov 26;8:215. [Version 1] doi: 10.15694/mep.2019.000215.1 (PMC10712491; doi:10.15694/mep.2019.000215.1)
Supplement: Peer review history

# Reviewer report: Reviewer response for version 1

**DOI:** 10.21956/mep.19945.r30283

**Published:** 2019-12-04

**Authors:** Trudie Roberts

**Affiliations:** 1 Leeds Institute of Medical Education

This review has been migrated. The reviewer awarded 4 stars out of 5 An increasingly important area so I applaud the desire to improve the education here. Understanding more about the actual on line course would be of interest. For example did you used videos or audio podcast like elements as well as normal screen learning. I agree that technological issues do need to be dealt with early as these learners are busy people

Reviewer Expertise:

NA

No decision status is available

# Reviewer report: Reviewer response for version 1

**DOI:** 10.21956/mep.19945.r30284

**Published:** 2019-11-27

**Authors:** BALAJI ARUMUGAM

**Affiliations:** 1 TAGORE MEDICAL COLLEGE AND HOSPITAL

This review has been migrated. The reviewer awarded 4 stars out of 5 Thanks for the invite to review the article. This research article and study design carries a good message that event with the smaller sample size, the qualitative studies can produce good results to assess the effectiveness of an educational tool. FGD was done with four group of residents on six themes. The thematic framework analysis was done clearly. Keep up the work. Congrats. One suggestion Word cloud image would have been better.

Reviewer Expertise:

NA

No decision status is available
